# A Condensed Concordance of the Homœopathic Materia Medica

**Published:** 1894-07

**Authors:** 


A CONDENSED CONCORDANCE OF THE HOMŒOPATHIC MATERIA MEDICA. By J. G. Malcolm, M. D. Published and sold by the author, 122 La Salle Street, Chicago, Illinois. Bound in sheep, $8.00; half morocco, $9.00.

Only the advance sheets of this important work lie before us. The whole work will be ready some time in June and will consist of one thousand pages.

The arrangement is by chapters. The chapter begins with symptomatology, relating to the particular organ to which the heading of the chapter refers.

This symptomatology is a series of paragraphs, each paragraph being a statement of symptoms occurring in the pathogenesis of a remedy the name of which stands at the head of that paragraph.

Then follows the repertory for the chapter; in which the rubrics are in alphabetical order, of course, and the names of the remedies are in two different kinds of type according to their value. Those in bold-faced type refer to the symptoms which are in *italics* in the symptomatolgy.

The crying need of the day is a good repertory. Dr. Malcolm's seems to be the latest and most convenient of the several noble works of that order which are already before the profession.

We cordially recommend *The Condensed Concordance* of Dr. Malcolm.

